# Public Health in Bristol, 1906-7

**Published:** 1907-12

**Authors:** D. S. Davies

**Affiliations:** Medical Officer of Health.


### PUBLIC HEALTH IN BRISTOL, 1906–7.

BY

D. S. DAVIES, M.D.,

*Medical Officer of Health.*

---

THE general health returns, which were very favourable for the year 1906, have continued to be satisfactory during the first three quarters of 1907.

*The recorded death-rate* for the year 1906 was 14.5 per 1,000, compared with a rate of 14.7 for the previous year, and with a rate for the seventy-six large towns of England and Wales of 15.9.

The rates for the four quarters of 1906, and for the three completed quarters of 1907, are here shown for comparison :—

| | 1906. | 1907. |
|---|---|---|
| 1st Quarter .. .. | 16.84 .. | 17.40 |
| 2nd Quarter .. | 13.66 .. | 12.58 |
| 3rd Quarter .. .. | 13.10 .. | 10.24 |
| 4th Quarter .. .. | 14.79 .. | — |

**The birth-rate** for 1906 was 25.8, a decrease on that of the previous year. This rate has shown an almost uninterrupted decline since 1882. The **annual marriage-rate** is also decreasing.

**Infant Mortality.**—The proportion of deaths of infants under one year to births in 1906 was 127 per 1,000, which is somewhat higher than that for the previous year (122). The highest rates were noted in Bristol Central, 173, St. Philip, 153, and the lowest in Ashley, 79. The general infantile rate is satisfactory, compared with 145 recorded in the seventy-six large towns of England and Wales, but the most crowded districts are susceptible of improvement.

The infant mortality rates for the four quarters of 1906, and for the three completed quarters of 1907, are here given for comparison :—

| | 1906. | 1907. |
|---|---|---|
| 1st Quarter .. .. | 148 .. | 118 |
| 2nd Quarter .. | 93 .. | 88 |
| 3rd Quarter .. .. | 156 .. | 99 |
| 4th Quarter .. .. | 111 .. | — |

The influence of a somewhat damp and cold summer and early autumn is apparent in the diminution shown in the third quarter of 1907, chiefly due to the small amount of infantile diarrhœa.

**The chief epidemic diseases.**—There has been no notable occurrence in regard to these diseases.

*Scarlet fever*, while still occurring in scattered cases, is generally of a mild type, and gives rise to little mortality.

*Diphtheria,* while distinctly less fatal than in the period 1900–04, still persists, and occasionally shows severe forms; but the tendency to form school outbreaks has declined, and children at susceptible ages appear to have acquired considerable immunity.

*Enteric fever.*—This disease only occurred in sporadic form in 1906, except in the Bristol Central District, where an outbreak centred round a shell-fish shop receiving supplies from a tidal river in South Wales. The type of case was severe, but the outbreak did not assume alarming dimensions.

A continuing outbreak at a "home" in the city is at present (November, 1907) engaging attention. For some months case after case has continued to crop up at intervals, in spite of every usual precaution. The continuance of infection has been clearly traced to milk, but the source of re-infection of this food has hitherto been non-apparent. It may possibly be due to a case of "carrier" typhoid, and careful observations are now in hand with a view to confirm or disprove this supposition.

*Small-pox.*—During the past few years small-pox has adopted the annual habit of paying the city a tentative spring visit. The type has continued to be very mild, leading to difficulty in tracing out contacts, and to increased chance of missed cases; but, on the other hand, the infectivity of this mild type appears to be comparatively small, and, as a fact, each outbreak has been readily repressed.

This disease, absent since June, 1905, re-appeared in January, 1906, lasting up to June, this period affording a scattered product of twenty-seven cases.[1]

The interest of the outbreak centred in its introduction and, commencing, spread in a public elementary school, a quite unique experience in Bristol. Investigation showed that three out of four children in one class in the school were unvaccinated. The obvious remedy soon stopped the spread; but the strictness of the conditions for success is shown by the necessity of having, at one time during the outbreak no less than six hundred persons under observation. This was over by the end of March.

[1] *Annual Report of Medical Officer of Health,* pp. 30–33.

A second introduction, apparently unconnected with this one, lasted from March to May, owing to two " missed " cases, but calls for no special comment.

From May, 1906, until January, 1907, no fresh introduction occurred ; in January, however, it re-appeared, and resulted in five cases, one fatal. This outbreak was blotted out by April, and no further introduction has taken place.

The summer session of Parliament was distinguished by the passing of some important Acts bearing on public health.

(1) *The Notification of Births Act,* 1907.—This is an adoptive Act, and its object is to secure notification of each birth within thirty-six hours to the medical officer of health, so that advice may be extended to the mother, where needed, as to the management and feeding of her infant. Adoption of the Act in any district is subject to the approval of the Local Government Board, who will require to be satisfied that the local authority will provide the necessary machinery, health visitors, &c., for utilising its provisions. Some opposition to the Act as it stands has been in some quarters expressed by the medical profession, who may be compelled to a violation of professional secrecy ; and in place of its adoption—at Gateshead, for example—a voluntary notification by the medical attendant has been substituted.

(2) The Education (Administrative Provisions) Act, 1907, provides, *inter alia,* that the powers and duties of a local education authority shall include the duty to provide for the medical inspection of children, immediately before, or at the time of, or as soon as possible after their admission to a public elementary school, and on such other occasions as the Board of Education directs ; and the power to make such arrangements as may be sanctioned by the Board of Education for attending to the health and physical condition of the children educated in public elementary schools.

The effect of this Act will be to make universal that medical inspection of school children which has proved advantageous in the few districts of England where it has been attempted, and to bring England into line with the advance in the direction for some years made in America and upon the Continent.

The proviso that in any exercise of powers under this section the local education authority may encourage and assist the establishment or continuance of voluntary agencies, and associate with itself representatives of voluntary associations for the purpose, is evidence of the value attached by the department to the assistance which voluntary endeavour, when started in a friendly spirit, can afford to public bodies in their executive work; assistance which is particularly helpful in matters relating to the care of the young, the assistance of mothers at and after child-birth, and the teaching of domestic hygiene.

*The Public Health Acts Amendment Act*, 1907, contains, in Parts III., IV. and V., forty-one sections, variously amending and supplementing previous Acts in regard to sanitary provisions, infectious diseases, and common lodging houses. The Act may be applied in part, or wholly, by an order of the Local Government Board to any sanitary district.

*The Public Health* (*Regulations as to Food*) *Act*, 1907.—This short act of three sections implies a good deal more than its length would suggest. It is entitled "An Act to enable regulations to be made for the prevention of danger arising to public health from the importation, preparation, storage, and distribution of articles of food." The regulations will be framed by the Local Government Board under the Public Health (Ports) Act, 1896, and will materially add to the executive duties and obligations of port sanitary authorities.

*The Cholera Regulations* of the Local Government Board, dated September 9th, 1907, reproduce the main provisions of previous Orders, with modifications and amendments in accordance with the decisions of the Paris Convention, and giving recognition to the recent developments of knowledge as to rat infection, plague, and mosquito infection in yellow fever. The Central Department is fully alive to the necessity for preparation in advance, especially in view of the possibility of renewed activity of cholera, and the port sanitary districts are to be maintained in a proper state of efficiency.

